# Cost-effectiveness of Ruxolitinib vs Best Available Therapy in the Treatment of Myelofibrosis in Spain

**DOI:** 10.36469/9808

**Published:** 2017-11-29

**Authors:** María Teresa Gómez-Casares, Juan Carlos Hernández-Boluda, Antonio Jiménez-Velasco, Joaquin Martínez-López, María Giovanna Ferrario, Irmina Gozalbo, Joana Gostkorzewicz, Rudi Subirá

**Affiliations:** 1 Department of Hematology Doctor Negrín University Hospital, Las Palmas de Gran Canaria, Spain; 2 Department of Hematology Clinic University Hospital, Valencia, Spain; 3 Department of Hematology and Hemotherapy Regional University Hospital, Málaga, Spain; 4 Department of Hematology Hospital Universitario 12 de Octubre, Madrid, Spain; 5 Outcomes’10, Castellón de la Plana, Spain; 6 Novartis Oncology, Barcelona, Spain

**Keywords:** janus kinase (jak) inhibitor, splenomegaly, myelofibrosis, jakavi® (ruxolitinib), cost-effectiveness

## Abstract

**Introduction:** Primary myelofibrosis (MF) is a rare hematologic disease belonging to the group of Philadelphia-negative chronic myeloproliferative neoplasms. Identification of the Janus Kinase (JAK) gene mutations inaugurated a new era in the targeted therapy of myeloproliferative diseases. Ruxolitinib is the first JAK1/JAK2 inhibitor specifically approved for the treatment of disease-related splenomegaly or symptoms in adult patients with primary myelofibrosis. The objective of this study was to assess the cost-effectiveness of ruxolitinib vs best available therapy (BAT) in MF patients in Spain.

**Methods:** A decision-tree and Markov model were adapted to the Spanish setting to assess the cost-effectiveness of ruxolitinib vs. BAT on a lifetime horizon (≤15 years) from the societal perspective, while healthcare system perspective was included in the one-way sensitivity analysis. The population was assumed to be similar to that of the COMFORT-II clinical trial (CT), which was also the source of treatment efficacy data. BAT composition was derived from the same CT and validated with Spanish experts. Utilities were derived from the COMFORT-I CT. Costs included treatment, management, hospitalizations, emergency and outpatient visits, as well as adverse events and end-of-life costs. Additionally, costs associated to productivity loss were taken into account. Resource use was validated with experts and costs were extracted from Spanish sources. A probabilistic sensitivity analysis was also performed to evaluate the consistency of the results under the uncertainty or variability of the input data.

**Results:** Patients on ruxolitinib accumulated 6.1 life years gained (LYGs), resulting in 73% extra life-years compared to patients treated with BAT (3.5LYs gained). Ruxolitinib provided 4.4 quality-adjusted life years (QALYs), with a 99% improvement compared to BAT (2.2 QALYs). This analysis gave an incremental cost of €47 199 per LYG and an incremental cost of €55 616 per QALY gained from the societal perspective.

**Conclusions:** Ruxolitinib would be cost-effective in Spain according to the end-of-life criteria defined by the NICE and commonly referred for Spain (cost-effectiveness threshold of €61 500/QALY), in line with results published for other European countries.

| This is an Open Access article under the CC BY 4.0 license.

## BACKGROUND

Primary myelofibrosis (MF) is a rare hematologic disease included in the class of Philadelphia-negative chronic myeloproliferative neoplasms. Its prevalence is generally established at 2/100 000 people, which yields an estimation of approximately 1400 patients in Spain.[1]

Identification of the Janus Kinase (JAK) gene mutations opened a new era in the targeted therapy of myeloproliferative diseases. Ruxolitinib is the first JAK1/JAK2 inhibitor specifically approved for the treatment of myelofibrosis-related splenomegaly or symptoms, owing to the evidence of rapid and sustained splenomegaly reduction, noticeable symptom improvement, and overall survival (OS) increase in randomized clinical trials.[2–5]

Recent national[6] and international[7,8] guidelines recommend the use of ruxolitinib for the first-line treatment of adult patients with primary or secondary MF presenting splenomegaly and/or constitutional symptoms regardless of the JAK2 V617F mutational status (grade of evidence 1A).[7]

The significant symptom burden associated to MF reflects on high healthcare costs. A recent north-American study showed that medical and pharmacological costs for patients with MF were 5-times higher than for a group of control patients without cancer,[9] reaching values as high as $34 690 (€25 972) per patient and year. Furthermore, a study carried out in Spain to estimate the indirect and non-medical costs associated to MF reported a mean cost of €86 315 per patient, which increased to €104 153 in patients still working, and €168 459 for more symptomatic patients.[1]

Considering evidence for long-term efficacy of ruxolitinib in patients with MF, this study aims to estimate the cost-effectiveness of ruxolitinib vs Best Available Therapy (BAT), i.e., a combination of treatments that are used to control MF-related symptoms although they're not specifically indicated for MF, from the societal perspective in Spain.[1]

## METHODS

A global model built in Microsoft Excel® was adapted to the Spanish National Healthcare System (NHS) setting. The model simulates the evolution of a cohort of 1000 patients through a set of three health states until a predefined lifetime horizon is reached, allowing the estimation of total costs and clinical benefits accumulated in accordance to the treatment arm to which patients are assigned.

### Structure

The model is structured in two main parts: a decision tree and a Markov model (Figure 1).

The decision tree assigns half of the cohort to the ruxolitinib treatment, and the other half to the BAT treatment. Once the treatment is assigned, patients enter the Markov model. There are three possible health states: alive on-treatment, alive off-treatment and dead, corresponding to patients that are alive and still on treatment, those who are alive but have withdrawn treatment and those who have deceased. Transition probabilities from the on-treatment to the off-treatment alive state are based on the "treatment discontinuation" curves of the COMFORT II clinical trial,[3] while the probability of transitioning from the alive states to the dead state are based on the OS curves.[10] Cycle length was 28 days.

**Figure 1.** Decision Tree and Markov Model Health States

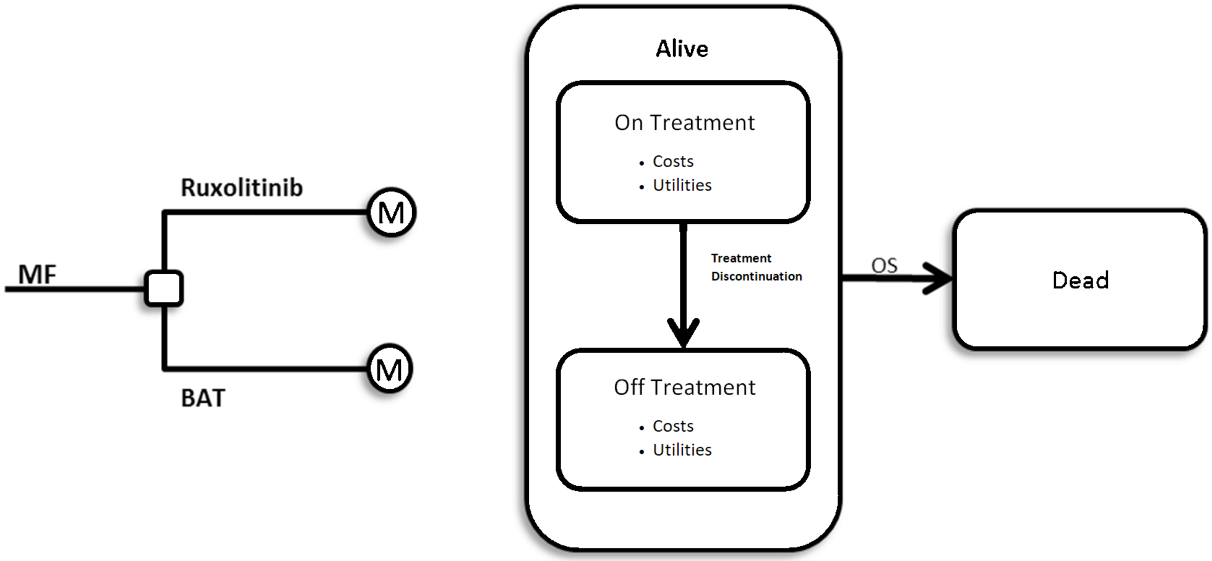

### Population

Clinical characteristics of the population were assumed to be as reported in the COMFORT II[3] clinical trial.

### Perspective

A societal perspective was adopted for the base case. However, the perspective of the Spanish NHS was considered as an alternative scenario in the sensitivity analysis by not including patients' productivity loss.

### Time Horizon

A lifetime horizon of 15 years was considered, based on the NICE ERG report[11] and according to the general life expectancy of patients.

### Discount Rate

A discount rate of 3% in both costs and clinical benefits was applied, being varied to a 0%-5% range in the sensitivity analysis.[12]

### Clinical Effectiveness

*Life Years Gained*

Life years (LY) gained are estimated as the time patients remain alive either on- or off- treatment. The transition probabilities are obtained from the OS curves of the COMFORT II-5 years[10] clinical trial, adjusted to account for the crossover between treatment arms using the RPSFT (rank-preserving structural failure time) method. To allow the extrapolation from the trial 5 years follow-up to the 15-year time horizon, the OS curve corresponding to the BAT treatment arm was parameterized using a lognormal distribution (according to the Bayesian Information Criteria). The OS curve of ruxolitinib was reconstructed using the Hazard Ratio (HR) obtained from the clinical trial.[10] Time on treatment from the COMFORT-II trial[3] was parameterized using a Gompertz distribution according to the same criteria.

Additionally, a 1.21% and 2.33% of patients in the ruxolitinib and BAT treatment arms respectively were assumed to undergo myelofibrosis transformation to acute myeloid leukemia (AML) as reported in the COMFORT-I 3 year analysis,[4] affecting quality of life and the corresponding treatment costs.

*Quality-adjusted Life Years Gained*

Both COMFORT trials assessed health related quality of life (HRQoL) as an exploratory endpoint, although HRQoL results from COMFORT-II trial were limited by missing data for many patients.[4,5] On the contrary, data for ruxolitinib patients in the COMFORT-I trial were reported for the majority of patients (136/155).[4]

Based on this, MF-8D utilities from the COMFORT-I clinical trial[4,13] were assigned to each health state and treatment arm to take into account the quality of life of patients.[13] Total quality-adjusted life years (QALYs) gained by a patient were estimated as the sum of the LY spent in each health state weighted by their utility.

**Costs**

Total costs were estimated as a sum of pharmacological, resource use, adverse-events (grade 3-4) management costs, loss of productivity, transformation to AML, and end-of-life costs. All costs are updated to €, 2016. BAT was considered as an aggregate of several treatments including antineoplastic agents, glucocorticoids, anti-anemic treatments, immunomodulatory treatments, interferons, purine analogs, hormone therapy, melphalan and cytarabine, allowing for polymedication as recommended by experts in the treatment of MF in Spain. As depicted in Table 1, approximately half of the treatments included in the BAT basket consisted of hydroxyurea, in accordance with data from COMFORT-II trial and expert validation.[5] Unit pharmacological costs for BAT were obtained from the Official Spanish Pharmacy database Bot Plus Web 14 and sum up to €16.66/day. The ex-factory price of ruxolitinib 15mg is €3583.33 per 56 pills,[14] which correspond to a daily cost of €127.98, considering a daily dose of 30 mg.[15] Thus, applying the mandatory 7.5% discount in force,[16] daily cost of ruxolitinib is equal to €118.38. Similarly, the ex-factory price with the corresponding mandatory discounts were applied to the BAT treatments.

**Table 1.** BAT Composition Assumed in the Model

| | Patients (%) | Source |
|---|---|---|
| Other antineoplastic agents | 50.7% | Expert consensus, 5 |
| Hydroxyurea | 46.6% | |
| Anagrelide | 5.5% | |
| Glucocorticoids | 22.5% | |
| Other antianemia preparations | 25% | |
| Other immunomodulatory agents | 2.7% | |
| Purine analogs | 5.5% | |
| Antigonadotropins and similar agents | 17.5% | |
| Interferons | 2.0% | |
| Nitrogen mustard analogs | 2.7% | |
| Pyrimidine analogs | 7.5% | |
| No Therapy | 32.9% | |
| BAT combined | 169.0%* | |

*The sum of percentages exceed 100% as polymedication was allowed.

Use of resources included monitoring of laboratory values, emergency visits, hospitalizations and outpatient visits. Unit costs were obtained from the eSalud Spanish database.[17] Frequency of use was extracted from the literature[18] and modified according to expert consensus when necessary (Table 2).

**Table 2.** Unit Cost, Frequency and Percentage of Patients Requiring Use of Resources

| | Patients requiring resources (%) | Frequency (times per year) | Frequency (times per cycle) | Unit cost (€) | Cost per cycle (€) | Source |
|---|---|---|---|---|---|---|
| Resource use | | | | | | |
| Monitoring of laboratory values | 100% | 11.02 | 0.85 | 4.99 | 4.24 | Expert consensus 17,18 |
| Emergency visit | 100% | 1* | 0.15 | 73.00 | 11.19 | |
| Hospital stay | 100% | 2* | 0.08 | 4753.93 | 364.44 | |
| Outpatient visit | 100% | 11.02* | 0.85 | 65.03 | 55.28 | |

*As per expert consensus.

Adverse events accounted for thrombocytopenia, anemia, pyrexia, and pneumonia, as reported in COMFORT-I[19] and validated by the experts. Unitary costs were obtained from the eSalud Spanish database (Table 3).[17] Costs associated to AML transformation and end-of-life costs are detailed in Table 3.

**Table 3.** Adverse Events, AML Transformation and End-of-life-related Costs

| | (%) with ruxolitinib | (%) with BAT | Frequency (times per 48 weeks)* | Frequency (times per cycle) | Unit cost (€) | Cost per cycle (€) | Source |
|---|---|---|---|---|---|---|---|
| **Grade 3-4 adverse event** | | | | | | | |
| Thrombocytopenia | 4.1% | 4.1% | 1 | 0.08 | 1404.62 | 117.05 | Expert consensus, 17,19 |
| Anemia | 30.0%† | 30.0%† | 1 | 0.08 | 1654.48 | 137.87 | |
| Pyrexia | 2.1% | 0.0% | 0.46† | 0.04† | 632.05 | 52.67 | |
| Pneumonia | 1.4% | 4.1% | 1 | 0.08 | 2688.66 | 224.05 | |
| **Other** | | | | | | | |
| AML transformation | 2.33% | 1.21% | - | - | 1864.82 | - | 4, 30 |
| End of life | - | - | - | - | 2657.90 | - | 4, 31 |

*Assumed one event per 48 weeks for both treatments as reported in COMFORT-II; †As per expert consensus.

Loss of productivity is incorporated in the model as productivity cost offset for each treatment arm. The cost offset is then subtracted from total costs at each cycle. Based on the age distribution of patients in the COMFORT-II clinical trial and the average annual income from the Spanish National Institute of Statistics (Table 4),[20] average annual income in the study cohort was estimated at €12 556 (€48.13/day). Mean number of worked days/year was estimated at 255 and 250 for ruxolitinib and BAT, respectively. This estimation was based on a report from Mesa et al.[21] in which a mean number of 0.9 missed days per month was observed in patients with myelofibrosis. A 45.9% fewer missed days of work was assumed for ruxolitinib arm based on the percentage of patients achieving ≥50% change from baseline in the MF-SAF TSS in COMFORT-I.[4] A proportion of 25.1% of patients was assumed to be working in both treatment arms.[21] Based on these data, productivity cost offset per cycle added up to €236.04 and €231.41 (€3018.64 and €3079.04/year) for ruxolitinib and BAT, respectively.

**Table 4.** Average Annual Income in the Study Cohort

| Age Groups in COMFORT-II | % Patients in Trial | Average Daily Income (€)* | Source |
|---|---|---|---|
| <45 | 2.7% | 89.76 | |
| 45-65 | 45.2% | 101.11 | |
| 66-75 | 41.1% | 0 | 3, 20 |
| >75 | 11.0% | 0 | |
| Total | 100% | 48.13 | |

*Assuming a maximum of 261 working days/year (considering a 5 day work week)

### Outcomes

Clinical outcomes of the model are LY and QALY gained. Economic outcomes are the total costs accrued by a patient from entrance to the model until the lifetime horizon is reached.

The incremental cost per LY gained (€/LY) and incremental cost per QALY gained (€/QALY) are estimated as the quotient between the incremental costs and the incremental QALY or LY of ruxolitinib vs BAT.

### Sensitivity Analysis

A One-way sensitivity analysis (OWSA) and a probabilistic sensitivity analysis (PSA) were run to evaluate the consistency of the results under the uncertainty or variability of the input data.

The OWSA sequentially introduces a variation of ±20% of the base case value for each of the input parameters with the exception of ruxolitinib and BAT costs, for which three additional price scenarios were included in the sensitivity analysis in which a reduction of 5%, 10%, and 15% when respect to the base case cost was considered. Finally, the payer's (Spanish NHS) perspective was considered as an alternative scenario in the sensitivity analysis, by not including the loss of productivity costs. Results were recorded and presented through a tornado diagram.

The PSA runs a second order Monte Carlo simulation with 1000 iterations while varying the input values according to a predefined probability distribution (gamma/beta) and its corresponding parameters. For the parameterized survival curves, a Cholesky decomposition of the variance/covariance matrix was used to vary the defining parameters.

## RESULTS

### Base Case

Overall, patients in the ruxolitinib treatment arm accumulated 6.1 LYs gained, resulting in 73% extra life-years as compared to patients treated with BAT (3.5 LYs gained). When adjusted by quality, ruxolitinib provided 4.4 QALYs, a 99% improvement compared to BAT (2.2 QALY) (Table 5).

**Table 5.** Clinical Benefit Estimated with Ruxolitinib and BAT Use

| Health State | Ruxolitinib | BAT | Incremental |
|---|---|---|---|
| **LY gained** | | | |
| On treatment | 3.25 | 0.96 | 2.29 |
| Off treatment | 2.84 | 2.55 | 0.29 |
| **Total** | **6.09** | **3.51** | **2.58** |
| **QALY gained** | | | |
| On treatment | 2.73 | 0.70 | 2.03 |
| Off treatment | 1.67 | 1.50 | 0.17 |
| Leukemic transformation | -0.01 | -0.01 | 0.00* |
| **Total** | **4.40** | **2.21** | **2.18** |

*Rounded value for 0.0009. BAT: best available treatment; QALY: quality adjusted life year

Total lifetime costs per patient in the ruxolitinib arm were €164 964, while only €43 425 with BAT (Table 6).

**Table 6.** Total Lifetime Costs Estimated with Ruxolitinib and BAT Use

| | **Costs (€)** | | |
|---|---|---|---|
| **Health State** | **Ruxolitinib** | **BAT** | **Incremental** |
| On treatment | 154 094 | 11 977 | 142 117 |
| Off treatment | 18 867 | 31 841 | - 12 973 |
| Leukemic transformation | 139 | 156 | - 17 |
| End of life | 1866 | 2348 | - 482 |
| Productivity loss (cost offset) | - 10 002 | - 2897 | - 7106 |
| **Total** | **164 964** | **43 425** | **121 539** |

BAT: best available treatment

As a result, the ICER of ruxolitinib vs BAT was €47 119/LYG, while the incremental cost per QALY gained was estimated in €55 616/QALY (Table 7).

**Table 7.** Incremental Cost per LY/QALY Gained of Ruxolitinib vs BAT

| | |
|---|---|
| Cost per LY Gained | €47 119 |
| Cost per QALY Gained | €55 616 |

QALY: quality adjusted life year

### Sensitivity Analysis

#### *One-way Sensitivity Analysis*

The OWSA showed that the most influent parameter was the OS curve lambda parameter for BAT, introducing a variation of +33%/-11% in the base case cost-effectiveness ratio (incremental cost/QALY) when varied a ±20%. The second most impacting variable was the baseline utility value obtained from the COMFORT-I study, which causes a +21%/-15% variation of the base case cost-effectiveness ratio when varied to its lower and upper value (Figure 2).

**Figure 2.** Tornado Diagram: ICER (cost/QALY) Variation Caused by Individual Variations of the Input Parameters

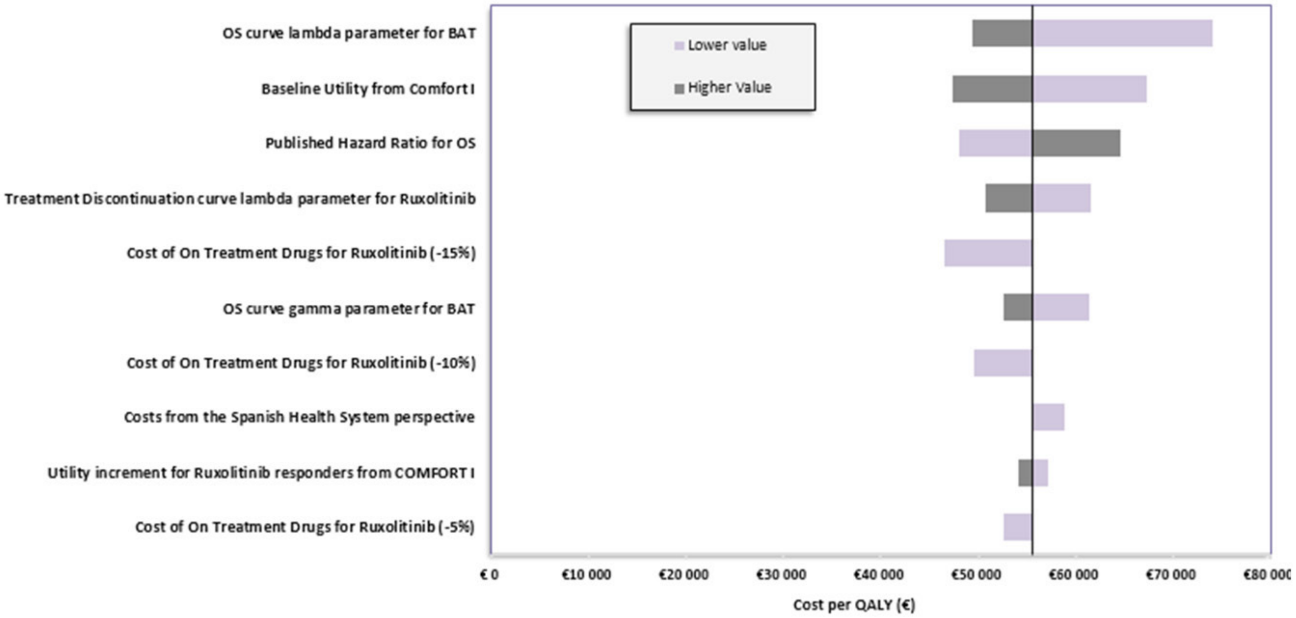

BAT: best available therapy; ICER: incremental cost-effectiveness ratio; QALY: quality adjusted life year; OS: overall survival

## Probabilistic Sensitivity Analysis

The PSA showed that 99.8% of the iterations fall into the upper-right quarter of the cost-effectiveness plane, meaning that ruxolitinib is more effective and more costly than BAT (Figure 3). Ruxolitinib has 61% probabilities of being cost-effective considering a willingness to pay (WTP) of €61 500/QALY (Figure 4).

**Figure 3.** Cost-effectiveness Scatterplot (Ruxolitinib vs BAT)

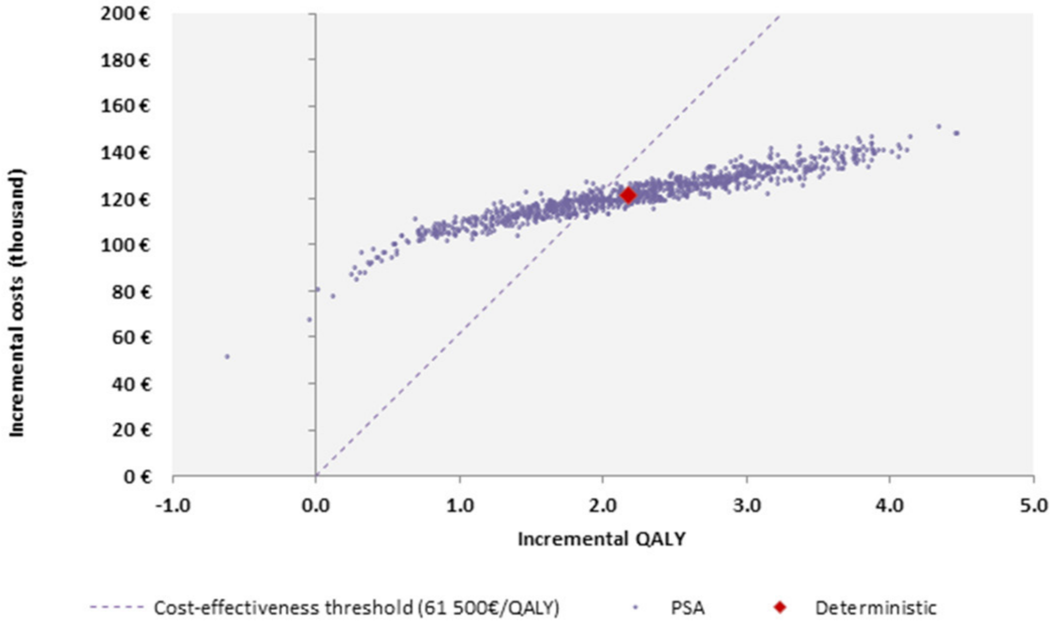

PSA: probabilistic sensitivity analysis; QALY: quality adjusted life year

**Figure 4.** Cost-effectiveness Curves (probability of being cost-effective according to the threshold)

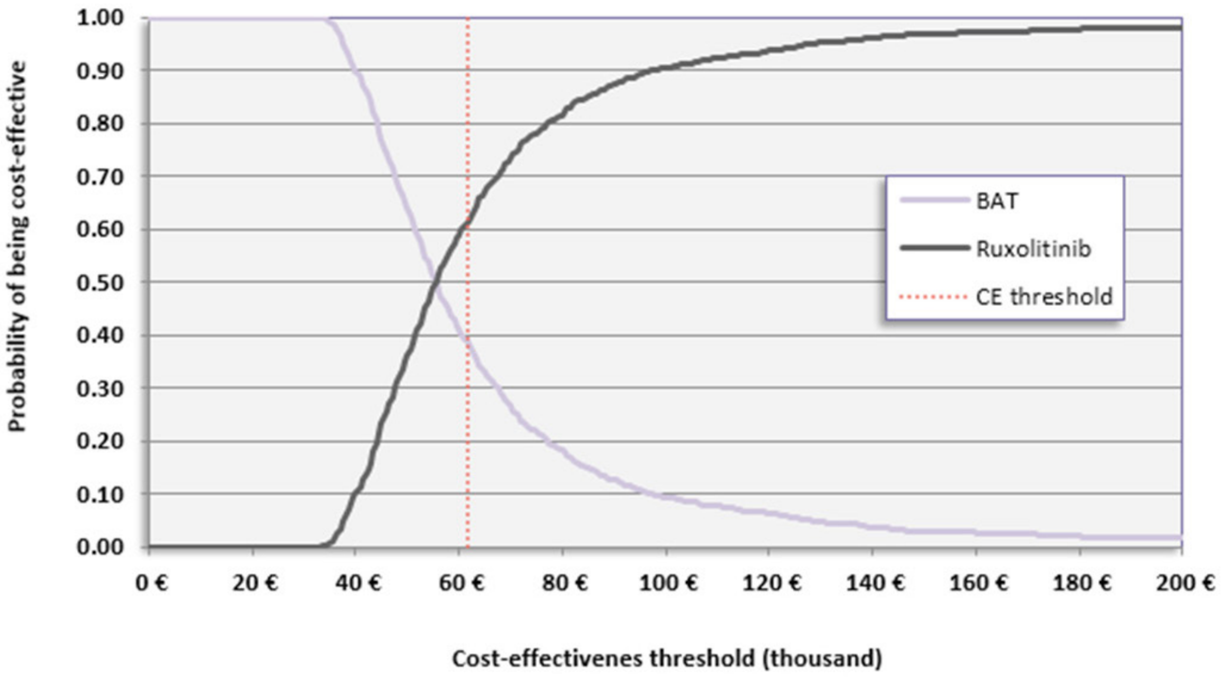

BAT: best available therapy; CE: cost-effectiveness

Discussion

This study estimates the cost-effectiveness of ruxolitinib vs BAT for the treatment of patients with myelofibrosis in the Spanish setting. This analysis gave an ICER of €47 119/LYG, while the incremental cost per QALY gained was estimated at €55 616/QALY. These values lie below the end-of-life (EoL) cost-effectiveness threshold defined by the NICE[22] and commonly referred for Spain (€61 500/QALY).

The cost-effectiveness of ruxolitinib has been previously evaluated in different settings.[2,11,23,24] In a study carried out in Portugal, Vandewalle et al.[2] estimated an increase of 2.43 LYs with ruxolitinib vs BAT with an incremental cost of €97 052, leading to an ICER of €40 000/LYG (year 2016). Based on this, authors concluded that ruxolitinib would be a cost-effective alternative in comparison with BAT in the treatment of MF in Portugal, considering a willingness to pay (WTP) of 50 000 €/LYG.[2] These results lie close to those achieved in the present analysis, mainly due to the similar OS-curve adjustment carried out in the analyses. Indeed, in both studies OS times for patients who had crossed over to ruxolitinib arm were corrected to account for the bias in survival time estimation for BAT.

In line with the Portuguese study, Hahl et al.[23] concluded that, in the Finnish setting, ruxolitinib treatment provides long terms benefits (2.43 QALYs) at a reasonable incremental cost (€102 802), yielding an ICER of €42 367/QALY. Thus considering a WTP of three times the local annual per capita gross domestic product (€114 705),[25,26] ruxolitinib may be considered cost-effective also from the Finnish health care perspective.

On the contrary, in an analysis conducted in the British setting and submitted to the NICE Evidence Review Group (ERG), ruxolitinib was associated with an increase of 1.04 LYs (1.15 QALYs) and an incremental cost of GBP 85 027 (€83 446) leading to an ICER of GBP 81 757/LY (€80 237/LYG) and of GBP 73 937/QALY (€72 562/QALY; year 2013). Based on these data, the NICE ERG concluded that ruxolitinib had demonstrated its efficacy in reducing splenomegaly and other MF related symptoms, but could not be deemed cost-effective from the National Health Service perspective.

In its appraisal, the ERG identified some weaknesses in the analysis submitted by the manufacturer. According to the committee, the long term extrapolation of efficacy data over the 35-year time horizon set in the analysis, would introduce considerable uncertainty in the results. Additionally, the ERG considered that long term survival data might have been confounded as no adjustment was performed to account for the crossover between treatments arms. Finally, the ERG found that, given the clinical relevance of leukemic transformation in patients with MF, transformation to AML should have been included in the base case analysis. The subsequent ERG review issued in March 2016,[27] accepted the cost-effectiveness of ruxolitinib vs BAT according to the EoL criterion. The estimated ICER for patients with intermediate-2 risk myelofibrosis was £26 000 per QALY gained (€24 882/QALY), and £38 000 per QALY gained (€36 367/QALY) for the high-risk subgroup. Although this only covers a part of the whole population that has indication for ruxolitinib treatment, the ERG estimated that the economic results were sufficiently robust to accept ruxolitinib as a cost-effective therapeutic alternative for myelofibrosis patients from the perspective of the British NHS.

The present model was improved to take into account criticisms conducted by the NICE ERG.[11] In particular, based on NICE recommendations, the time horizon of the analysis was reduced from 35 to 15 years, thus reducing the uncertainty introduced by the extrapolation of the efficacy data and providing more robust results. In addition, to further reduce uncertainty regarding treatment efficacy, OS-curve adjustment was carried out by means of RPSFT, thereby controlling the possible bias introduced by the crossover between BAT and ruxolitinib arm. Finally, rates of leukemic transformation from the COMFORT-I trial were used to predict the proportion of patients transforming to AML for each treatment arm and impact of these events on both costs and morbidity was estimated in the study population.

It is important to note that differently from previously published analysis, the present study adopt a societal perspective for the estimation of ruxolitinib cost-effectiveness in the base case scenario, allowing to capture the effect of treatment on patients productivity. This is particularly relevant considering that a significant proportion of MF patients are under 65 years old and still professionally active. Additionally, it has been shown that indirect costs of MF in Spain, including both the patients' productivity loss and the cost of informal care, sum up to an average of €15 142 per patient and year, thus highlighting the need of alleviating the symptom burden and improving HR-QoL of MF patients.[1]

A strength of this analysis is the use of the COMFORT-I utility data,[13] which were estimated using a specific health-related quality of life (HR-QoL) questionnaire to assess the improvement of the MF-related symptoms, in line with the FDA guidance.[28] Thus, the clinical trial included as a secondary endpoint the reduction of MF-related symptoms by ≥50%, showing that the proportion of patients achieving this target was significantly superior in the cohort of ruxolitinib as compared to the placebo cohort.[13] An independent data analysis showed that patients left untreated, ie, in treatment with placebo, experienced spleen volume and palpable length increase in most patients, with worsening of symptom score on all used PRO scales. These results demonstrated the progressive and debilitating effects of MF and the need of an effective spleen reduction treatment.[29]

This study must be read in the frame of its limitations. Mainly it must be noted that there is only little information published about MF in Spain, so the local resource use was difficult to define. To fill this gap, the adaptation of the model to the Spanish setting and input validation were carried out with the collaboration of a panel of four hematologists with wide experience in treating patients with MF in Spain. Also, BAT composition and dosage were refined with their help to make sure that they were representative of the Spanish clinical practice. Thus, despite this limitation, the use of validated input data together with the reduction of time horizon and the adjustment of the OS curves, sensibly contributed to the robustness of the results presented, as demonstrated by the sensitivity analyses performed.

## CONCLUSION

Our results suggests that, despite being more costly, treatment with ruxolitinib is associated with substantial long-term benefits in terms of OS, providing 2.5 extra LYs (6.1 vs 3.5) and double QALYs (4.4 vs 2.2) as compared to BAT, in patients with MF. Additionally, ruxolitinib has been shown to notably improve MF-related symptoms, which is reflected in this analysis by the fact that its acquisition cost is partly balanced by an increased productivity of the population.

Taking into consideration a WTP of €61 500/QALY adopted by the NICE and commonly referred for Spain in the evaluation of end-of-life treatments, it is possible to conclude that ruxolitinib provides good value for money compared to BAT in the treatment of MF-related symptoms in Spain.
